# Higher baseline platelet and preoperative platelets to lymphocytes ratio was associated with a higher incidence of axillary node pathologic complete response after neoadjuvant chemotherapy in HER2-low breast cancer: a retrospective cohort study

**DOI:** 10.3389/fonc.2025.1437677

**Published:** 2025-03-14

**Authors:** Shuai Yang, Guanying Liang, Junyi Sun, Lingbing Yang, Zitong Fu, Wantong Sun, Bo Wei, Abiyasi Nanding, Qin Wang, Shouping Xu

**Affiliations:** ^1^ Department of Breast Surgery, Harbin Medical University Cancer Hospital, Harbin, China; ^2^ Department of Pathology, Harbin Medical University Cancer Hospital, Harbin, China; ^3^ Department of Urology Xiang Ya Hospital, Central South University, Changsha, Hunan, China

**Keywords:** breast cancer, HER2-low, axillary node, neoadjuvant chemotherapy, pathologic complete response

## Abstract

**Background:**

HER2 expression has a central role in breast cancer carcinogenesis and is associated with poor prognosis. Lately, identification of HER2-low breast cancer has been proposed to select patients for novel HER2-directed chemotherapy and includes cancers with immunohistochemistry (IHC) 1+or 2+with negative fluorescence *in situ* hybridization (FISH), encompassing approximately 55–60% of all breast carcinomas. Neoadjuvant chemotherapy(NAC) is an important therapeutic modality for HER2-low breast cancer (BC). Immune inflammatory biomarkers have been reportedly linked to the prognosis of some different breast cancer types, with varying results. In this study, we investigated the possible predictive roles of blood-based markers and clinicopathologic features in axillary pathologically complete response (pCR) after neoadjuvant treatment (NAT) in HER2-low BC.

**Methods:**

HER2-low BC patients diagnosed and treated in the Harbin Medical University Cancer Hospital from January 2012 to December 2018 were included. Relevant clinical and pathological characteristics were included, and baseline and preoperative complete blood cell counts were evaluated to calculate four systemic immune-inflammatory markers: neutrophil-to-lymphocyte ratio (NLR), platelet-to-lymphocyte ratio (PLR), lymphocyte-to-monocyte ratio (LMR), and systemic immune-inflammation index (SII). The optimal cutoff values for these markers were determined using ROC curves and patients were classified into high-value and low-value groups based on these cutoff values. Univariate and multivariate logistic regression analyses were conducted to analyze factors influencing axillary pCR. The factors with independent predictive value were used to construct a forest map.

**Results:**

A total of 998 patients were included in the study. 35.6% (355 of 998) of patients achieved axillary pCR after NAC. The result of multivariate logistic regression analysis showed that Estrogen receptor (ER) (OR=2.18; 95% CI 1.43-3.32; P<0.001),pathology type (OR=0.51; 95% CI 0.40-0.65; P<0.001),baseline platelet (OR=1.45; 95% CI 1.02-2.05; P=0.037),preoperative PLR (OR=1.63; 95% CI 1.01-2.64; P=0.046) were significant independent predictors of ypN0.

**Conclusion:**

The forest map for predicting axillary pCR incorporates four variables, including ER, pathology type, platelet, platelet-to-lymphocyte ratio (PLR). In patients treated with NAC, a higher baseline platelet and a higher preoperative PLR was associated with a higher incidence of axillary pCR.

## Introduction

Breast cancer is a malignant tumor with high heterogeneity, and it presents a significant threat to women’s health (1, 2). Human epidermal growth factor receptor 2 (HER2) is a member of the human tyrosine kinase receptor family and serves as a crucial molecular marker for the diagnosis and treatment of breast cancer (3, 4). HER2-low breast cancer is defined as tumors with a HER2 IHC score of 2+ and negative FISH or IHC 1+ (5) and is found in approximately 60% of hormone receptor-positive (HR+) tumors and 40% of triple-negative breast cancer (TNBC) cases (6).

Compared with conventional breast cancer treatment, HER2-low expression breast cancer still uses traditional surgical resection (7). Preoperative neoadjuvant chemotherapy (NAC) is an important treatment that can reduce the stage of the tumor, increasing the chance of surgery (8). pCR in breast tumors is associated with improved prognosis and is an independent predictor of survival (9). At present, several studies have found that the pCR rate of HER2-low breast cancer patients after neoadjuvant therapy is between 13% and 30% (6, 10, 11). However, these data lack hematologic parameters for studies of pCR after neoadjuvant therapy.

Systemic inflammation plays a key role in tumorigenesis and cancer progression by stimulating angiogenesis, influencing immune surveillance and therapeutic efficacy (12), and promoting a favorable tumor microenvironment for cancer cell growth and spread (13).Several inflammation-associated hematological parameters, including the neutrophil-to-lymphocyte ratio (NLR), platelet-to-lymphocyte ratio (PLR) and lymphocyte-to-monocyte ratio (LMR)and systemic immune-inflammation index (SII), have been investigated as effective markers for predicting immunotherapy efficacy and patient prognosis with advanced BC (14–18). However, the predictive roles of these routinely available peripheral blood markers in patients with HER2-low BC receiving neoadjuvant chemotherapy has not been reported.

This study aims to investigate the predictive role of neoadjuvant chemotherapy in patients with HER2-low BC clinicopathologic features, baseline and preoperative NLR,PLR,LMR and SII axillary pCR. We found that higher baseline platelet and higher preoperative PLR were associated with a higher incidence of axillary pCR in patients with low HER2 expression. At the same time, we analyzed the effects of baseline and preoperative NLR, LMR, SII, baseline PLR and baseline neutrophils on axillary pCR. However, the P-values of these indicators were greater than 0.05, so there was no correlation between these indicators and axillary PCR rate.

## Materials and methods

### Patient selection and data collection

We retrospectively reviewed the clinical records in our institute from January 2012 to December 2018. The medical records of these patients were carefully reviewed to gain a comprehensive understanding of the effectiveness of breast cancer treatment and its associated factors. The inclusion criteria were as follows (1): Patients aged over 18 years. (2) Patients with HER2-0 or HER2-low (IHC 1+ or IHC 2+ and FISH-), breast cancer complete at least 4 cycles of anthracycline-taxeme-based NAC regimens.(3) Patients with a confirmed pathological diagnosis, complete clinical data, and peripheral blood indicators. (4) completed surgical treatment in our hospital with a pathological report. The exclusion criteria were as follows:(1) Patients who had distant metastasis at the time of diagnosis or bilateral breast cancer were excluded. (2) Patients who developed liver or kidney dysfunction during treatment and were unable to tolerate the treatment. (3) Patients with autoimmune diseases or other diseases that could affect peripheral blood indicators. A total of 998 patients were included in the study (**Supplementary Table 1**).

### Data extraction and assessment

Peripheral blood samples were collected at baseline (defined the first blood collection time for patients just admitted to the hospital) and before surgery (defined a second blood collection after at least four cycles of neoadjuvant chemotherapy is performed before the patient has surgery). The NLR was defined as the absolute neutrophil count divided by the absolute lymphocyte count. The PLR was defined as the absolute platelet count divided by the absolute lymphocyte count and the LMR was defined as the absolute lymphocyte count divided by the absolute monocyte count. The SII is defined as absolute neutrophil count multiplied by absolute neutrophil count divided by absolute lymphocyte count. Data that may affect treatment efficacy were also collected including age, ER, PR, HER-2 expression, pN, pathological type, Histological Grade, Ki67% and Molecular subtype.

Breast tumor pCR was defined as the absence of invasive carcinoma residue. Axillary lymph node pCR was defined as the absence of cancer in all dissected axillary lymph nodes.

### Chemotherapy regimens

Our study took anthracycline- (A-) based and/or taxane- (T-) based NAC regimens, repeat the cycle every three weeks for the selected regimens. All patients underwent a minimum of four cycles of NAC, antracycline and taxane regimens include: AC-T regimen: anthracyclines 100mg/m2, cyclophosphamide (C) 600mg/m2, followed by docetaxel 80-100mg/m2; TAC regimen: taxanes 75mg/m2, anthracyclines 50mg/m2, and cyclophosphamide 500mg/m2; AT regimen: taxanes 75mg/m2 and doxorubicin 60mg/m2.

Other regimens include: AC regimen: anthracyclines 90mg/m2 and cyclophosphamide 600mg/m2; TC regimen: docetaxel 80-100mg/m2 and cyclophosphamide 600mg/m2.

### Grouping criteria

According to postoperative axillary lymph node pathologic response assessment, all included patients were dichotomized into two groups: pCR and non-pCR groups. The demographic,clinical and hematologic characteristics were compared between the two groups.

### Statistical analysis

All statistical analyses were performed using IBM SPSS Statistics 26 software (https://www.ibm.com/cn-zh/spss). The data visualization was performed using R 4.3.0 (https://www.r-project.org/).

Categorical variables were expressed as numbers and percentages and were analyzed using the chi-square test. Normal distribution was assessed using the Shapiro-Wilk test. Normally distributed continuous variables were expressed as mean ± standard deviation (SD) and analyzed using the two-sample t-test, while non-normally distributed continuous variables were expressed as median and inter quartile range (IQR) and analyzed using the Mann-Whitney test.

A receiver operating characteristic (ROC) curve was constructed to estimate the optimal cut-off values for the baseline and preoperative NLR,PLR,MLR and SII, The cut-off values were selected based on the You-den index, which is calculated by subtracting 1 from the sum of sensitivity and specificity. Subsequently, the markers were further analyzed by dividing them into high and low values based on the optimal cut-off values. Logistic regression analysis was employed to conduct univariate analysis of the factors influencing pCR. The odds ratios (ORs) and 95% confidence intervals (CIs) were estimated for each variable. Subsequently, variables with a significance level of *P*<0.05 in the univariate analysis were included as potential predictors of pCR. *P*<0.05 is considered to indicate statistical significance for the difference.

## Results

### Patient and cancer characteristics

A total of 998 patients were included in the study. The mean age at initial diagnosis was 50 years (range 23-73). **Table 1** presents the baseline characteristics of the patients. The majority of patients are ER receptor positive, PR receptor positive (72.7%, 62.5%), HER2-0 (53.1%), HER2-1+ (41.4%), HER2-2+/FISH- (5.5%) and nearly 65% of patients have positive axillary lymph nodes. 78% of patients had invasive ductal carcinoma, and another 2.2% had unknown pathological type. Histology II (61.6%) and III (10.4%) were the most common grade histology, with 23.8% of histological grades unknown. A larger proportion of patients (72.3%) had a Ki67 proliferation index of ≥14%. Nearly 30% of patient’s molecular subtype belongs Luminal A, TNBC and 40% belongs Luminal B. A total of 355(35.6%) patients achieved Axillary pCR after NAT.

**Table 1:** Patient and cancer characteristics of HER2-0 and HER2-low patients.

Characteristics, n (%)	n = 998
Age	
≦50	529 (53.0)
>50	469 (47.0)
ER	
<1%	272 (27.3)
≧1%	726 (72.7)
PR	
<1%	384 (38.5)
≧1%	614 (61.5)
HER2	
0	530 (53.1)
1+	413 (41.4)
2+/FISH-	55 (5.5)
pN	
N0	355 (35.6)
N1	331 (33.2)
N2	205 (20.5)
N3	107 (10.7)
Pathology Type	
Invasive ductal carcinoma	778 (78.0)
Invasive breast cancer	189 (18.9)
Specific breast cancer	9 (0.9)
unknow	22 (2.2)
Histological Grade	
I	41 (4.1)
II	615 (61.6)
III	104 (10.4)
unknow	238 (23.8)
Ki67	
≦14%	276 (27.7)
>14%	722 (72.3)
Molecular subtype	
Luminal A	296 (29.7)
Luminal B	443 (44.4)
TNBC	259 (26.0)
Axillary node pathologic state	
pCR	355 (35.6)
Non-pCR	643 (64.4)

### Hematologic parameters

Peripheral blood samples were collected within 7 days before the first treatment and within 7 days before surgery **Table 2**, higher baseline neutrophil count (*P* = 0.017), higher baseline PLR count (*P* = 0.019), higher baseline and preoperative NLR (*P* = 0.005 and 0.028, respectively) and higher baseline and preoperative SII (*P* = 0.002 and 0.024, respectively) were observed in the non-pCR group than in the pCR group. However, no significant differences were observed between the two groups with respect to other hematological parameters.

**Table 2:** Hematological parameters of the patients.

Characteristics	pCR (355)	Non-pCR (643)	*p*
Baseline lymphocyte	1.97 (1.64-2.41)	1.95 (1.59-2.42)	0.497
Baseline neutrophil	3.75 (2.88-4.77)	3.92 (3.14-4.90)	0.017
Baseline monocyte	0.40 (0.31-0.49)	0.40 (0.32-0.49)	0.212
Baseline platelet	257 (216-299)	263 (226-304)	0.072
Baseline NLR	1.85 (1.44-2.51)	1.96 (1.54-2.70)	0.005
Baseline PLR	127.68 (100.91-163.68)	133.14 (104.20-169.35)	0.019
Baseline LMR	5.23 (3.96-6.72)	5.02 (3.83-6.36)	0.667
Baseline SII	467.43 (352.00-674.17)	509.00 (373.39-724.39)	0.002
Preoperative lymphocyte	1.41 (1.14-1.75)	1.39 (1.12-1.72)	0.787
Preoperative neutrophil	3.58 (2.74-4.87)	3.88 (2.82-5.16)	0.088
Preoperative monocyte	0.51 (0.39-0.66)	0.52 (0.40-0.70)	0.171
Preoperative platelet	276.00 (227.00-336.00)	274.00 (233.00-331.00)	0.951
Preoperative NLR	2.67 (1.95-3.61)	2.73 (1.95-3.94)	0.028
Preoperative PLR	200.55 (152.49-257.29)	202.14 (152.20-260.96)	0.368
Preoperative LMR	2.75 (2.10-3.81)	2.68 (1.94-3.59)	0.762
Preoperative SII	742.06 (517.69-1073.12)	773.75 (517.24-1143.64)	0.024

### Association of baseline hematological parameters with pCR

Based on the outcome variable pCR, ROC analysis was conducted for hematological markers to select the optimal cutoff values.

**Figure 1:**
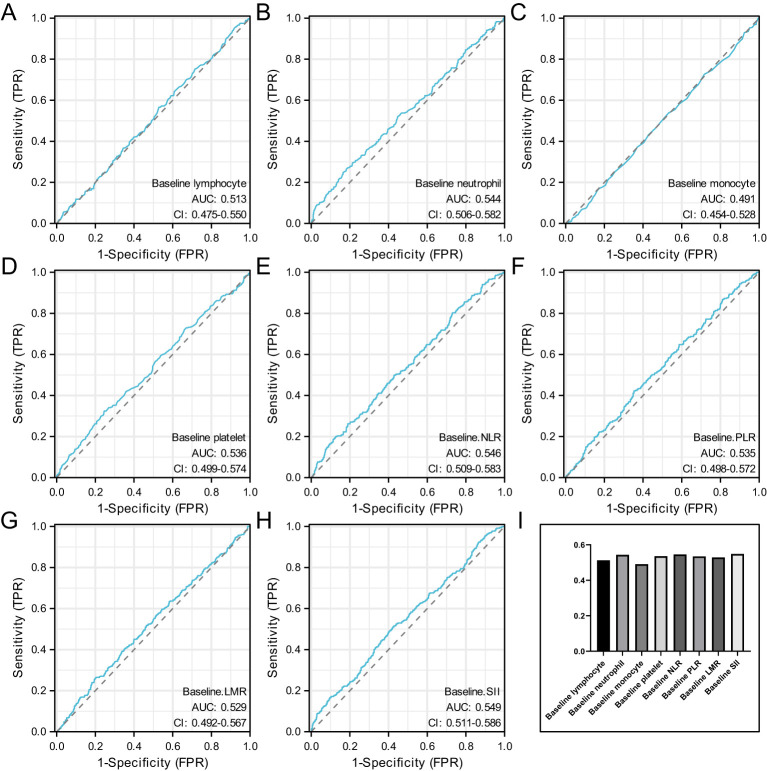
Represents the ROC curve for baseline lymphocyte **(A)**, represents the ROC curve for baseline neutrophil **(B)**, represents the ROC curve for baseline monocyte **(C)**, represents the ROC curve for baseline platelet **(D)**, represents the ROC curve for baseline NLR **(E)**, represents the ROC curve for baseline PLR **(F)**, represents the ROC curve for baseline LMR **(G)**, represents the ROC curve for baseline SII **(H)** and the AUC value of baseline hematological parameters **(I)**.

**Figure 1** shows the ROC curves for baseline hematological parameters. **Figure 1A** represents the ROC curve for baseline lymphocyte, with an AUC of 0.513 and an optimal cutoff of 1.895. **Figure 1B** represents the ROC curve for baseline neutrophil, with an AUC of 0.544 and an optimal cutoff of 2.875. **Figure 1C** represents the ROC curve for baseline monocyte, with an AUC of 0.491 and an optimal cutoff of 0.325. **Figure 1D** represents the ROC curve for baseline platelet, with an AUC of 0.536 and an optimal cutoff of 225.5. **Figure 1E** represents the ROC curve for baseline NLR, with an AUC of 0.546 and an optimal cutoff of 1.30. **Figure 1F** represents the ROC curve for baseline PLR, with an AUC of 0.535 and an optimal cutoff of 116.97. **Figure 1G** represents the ROC curve for baseline LMR, with an AUC of 0.529 and an optimal cutoff of 6.66. **Figure 1H** represents the ROC curve for baseline SII, with an AUC of 0.549 and an optimal cutoff of 468.09.

When the AUC value of baseline hematological parameters was greater than 0.05, indicating that these indicators were more closely related to axillary PCR before neoadjuvant therapy. **Figure 1I**.

### Association of preoperative hematological parameters with pCR

**Figure 2:**
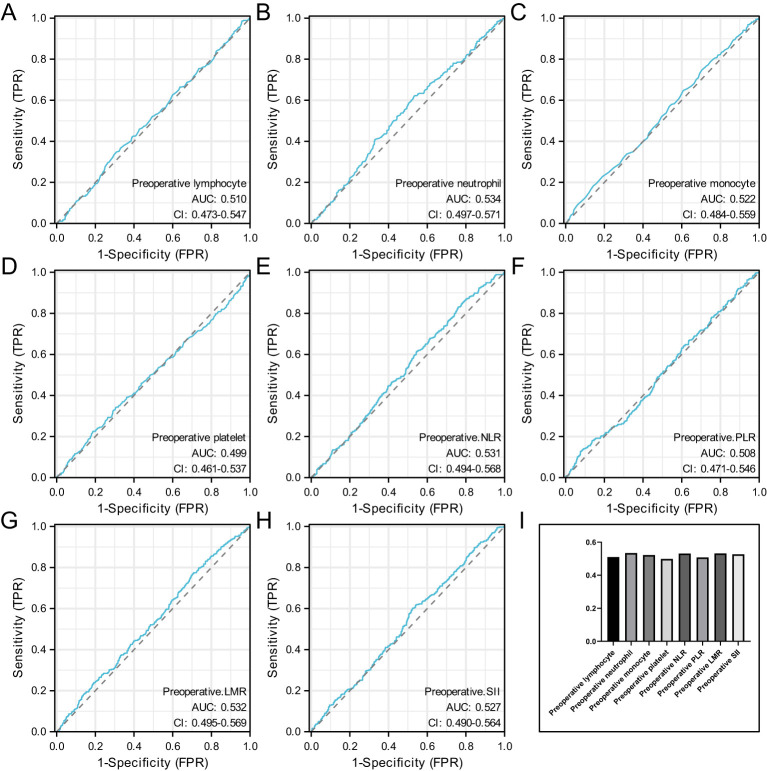
Represents the ROC curve for preoperative lymphocyte **(A)**, represents the ROC curve for preoperative neutrophil **(B)**, represents the ROC curve for preoperative monocyte **(C)**, represents the ROC curve for preoperative platelet **(D)**, preoperative the ROC curve for baseline NLR **(E)**, represents the ROC curve for preoperative PLR **(F)**, represents the ROC curve for preoperative LMR **(G)**, represents the ROC curve for preoperative SII **(H)** and the AUC value of preoperative hematological parameters **(I)**.

**Figure 2** shows the ROC curves for preoperative hematological parameters. **Figure 2A** represents the ROC curve for preoperative lymphocyte, with an AUC of 0.51 and an optimal cutoff of 1.635. **Figure 2B** represents the ROC curve for preoperative neutrophil, with an AUC of 0.534 and an optimal cutoff of 4.035. **Figure 2C** represents the ROC curve for preoperative monocyte, with an AUC of 0.522 and an optimal cutoff of 0.655. **Figure 2D** represents the ROC curve for preoperative platelet, with an AUC of 0.499 and an optimal cutoff of 349.5. **Figure 2E** preoperative the ROC curve for baseline NLR, with an AUC of 0.531 and an optimal cutoff of 4.00. **Figure 2F** represents the ROC curve for preoperative PLR, with an AUC of 0.508 and an optimal cutoff of 117.07. **Figure 2G** represents the ROC curve for preoperative LMR, with an AUC of 0.532 and an optimal cutoff of 2.07. **Figure 2H** represents the ROC curve for preoperative SII, with an AUC of 0.527 and an optimal cutoff of 835.04.

When the AUC value of preoperative hematological parameters was greater than 0.05, indicating that these indicators were more closely related to axillary PCR after neoadjuvant therapy. **Figure 2I**.

### Univariate analysis of axillary pCR

The proportions of patients achieving pCR based on different clinical and tumor characteristics are presented in **Table 3**. In univariate logistic regression, patients with ER≥1%, PR≥1%, Ki67>14% were more likely to achieve pCR compared to those with ER<1%(*P <*0.001), PR<1% (*P <*0.001), Ki67 ≤ 14% (*P* = 0.006). pCR is more readily available to patients with invasive ductal carcinoma (*P* = 0.000), patients with higher values of baseline neutrophil, baseline platelet, baseline NLR, baseline PLR, preoperative PLR and preoperative LMR were more likely to achieve pCR compared to patients with lower values (*P* = 0.002; *P* = 0.01; *P* = 0.004; *P* = 0.036; *P* = 0.012; *P* = 0.03 respectively). On the other hand, patients with lower values of baseline LMR, baseline SII, preoperative neutrophil, preoperative NLR, preoperative SII were more likely to achieve pCR compared to patients with higher values (*P* = 0.031; *P* = 0.009; *P* = 0.009; *P* = 0.012; *P* = 0.022 respectively).

**Table 3:** Univariate analysis for axillary pCR.

Characteristics	Overall	pCR	Non-pCR	*p*
	998	355	643	
Age				
≦50	529 (53.0)	192 (54.1)	337 (52.4)	0.659
>50	469 (47.0)	163 (45.9)	306 (47.6)	
ER				
<1%	272 (27.3)	143 (40.3)	129 (20.1)	<0.001
≧1%	726 (72.7)	212 (59.7)	514 (79.9)	
PR				
<1%	384 (38.5)	175 (49.3)	209 (32.5)	<0.001
≧1%	614 (61.5)	180 (50.7)	434 (67.5)	
HER2				
0	530 (53.1)	195 (54.9)	335 (52.1)	0.557
1+	413 (41.4)	139 (39.2)	274 (42.6)	
2+/FISH-	55 (5.5)	21 (5.9)	34 (5.3)	
Pathology Type				
Invasive ductal carcinoma	778 (78.0)	236 (66.5)	542 (84.3)	<0.001
Invasive breast cancer	189 (18.9)	97 (27.3)	92 (14.3)	
Specific breast cancer	9 (0.9)	5 (1.4)	4 (0.6)	
unknow	22 (2.2)	17 (4.8)	5 (0.8)	
Histological Grade				
I	41 (4.1)	16 (4.5)	25 (3.9)	0.146
II	615 (61.6)	174 (49.0)	441 (68.6)	
III	104 (10.4)	37 (10.4)	67 (10.4)	
unknow	238 (23.8)	128 (36.1)	110 (17.1)	
Ki67				
≦14%	276 (27.7)	79 (22.3)	197 (30.6)	0.006
>14%	722 (72.3)	276 (77.7)	446 (69.4)	
Molecular subtype				
Luminal A	296 (29.7)	92 (25.9)	204 (31.7)	<0.001
Luminal B	443 (44.4)	126 (35.5)	317 (49.3)	
TNBC	259 (26.0)	137 (38.6)	122 (19.0)	
Baseline lymphocyte				
low	438 (43.9)	152 (42.8)	286 (44.5)	0.66
high	560 (56.1)	203 (57.2)	357 (55.5)	
Baseline neutrophil				
low	194 (19.4)	88 (24.8)	106 (16.5)	0.002
high	804 (80.6)	267 (75.2)	537 (83.5)	
Baseline monocyte				
low	279 (28.0)	97 (27.3)	182 (28.3)	0.797
high	719 (72.0)	258 (72.7)	461 (71.7)	
Baseline platelet				
low	273 (27.4)	115 (32.4)	158 (24.6)	0.01
high	725 (72.6)	240 (67.6)	485 (75.4)	
Baseline NLR				
low	151 (15.1)	70 (19.7)	81 (12.6)	0.004
high	847 (84.9)	285 (80.3)	562 (87.4)	
Baseline PLR				
low	377 (37.8)	150 (42.3)	227 (35.3)	0.036
high	621 (62.2)	205 (57.7)	416 (64.7)	
Baseline LMR				
low	776 (77.8)	262 (73.8)	514 (79.9)	0.031
high	222 (22.2)	93 (26.2)	129 (20.1)	
Baseline SII				
low	452 (45.3)	181 (51.0)	271 (42.1)	0.009
high	546 (54.7)	174 (49.0)	372 (57.9)	
Preoperative lymphocyte				
low	677 (67.8)	232 (65.4)	445 (69.2)	0.239
high	321 (32.2)	123 (34.6)	198 (30.8)	
Preoperative neutrophil				
low	562 (56.3)	220 (62.0)	342 (53.2)	0.009
high	436 (43.7)	135 (38.0)	301 (46.8)	
Preoperative monocyte				
low	717 (71.8)	265 (74.6)	452 (70.3)	0.165
high	281(28.2)	90(25.4)	191(29.7)	
Preoperative platelet				
low	797 (79.9)	275 (77.5)	522 (81.2)	0.187
high	201 (20.1)	80 (22.5)	121 (18.8)	
Preoperative NLR				
low	784 (78.6)	295 (83.1)	489 (76.0)	0.012
high	214 (21.4)	60 (16.9)	154 (24.0)	
Preoperative PLR				
low	94 (9.4)	45 (12.7)	49 (7.6)	0.012
high	904 (90.6)	310 (87.3)	594 (92.4)	
Preoperative LMR				
low	273 (27.4)	82 (23.1)	191 (29.7)	0.03
high	725 (72.6)	273 (76.9)	452 (70.3)	
Preoperative SII				
low	566 (56.7)	219 (61.7)	347 (54.0)	0.022
high	432 (43.3)	136 (38.3)	296 (46.0)	

### Multivariate analysis of axillary pCR

**Figure 3:**
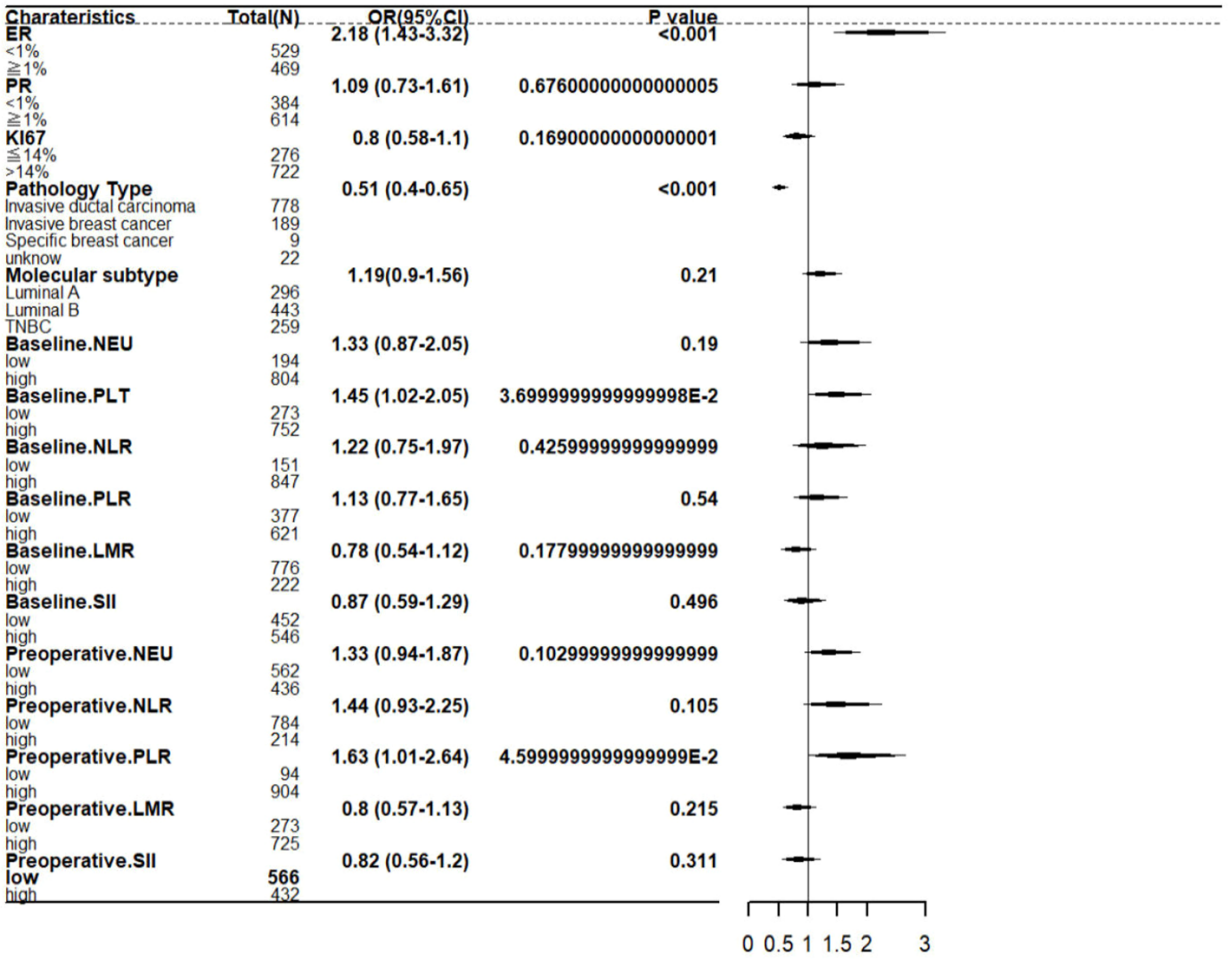
A forest map for axillary pCR based on independent influencing factors.

In the multivariate analysis, after including variables with P values<0.05 from the univariate analysis, two clinical and tumor characteristics and two hematologic markers still maintained their independent predictive roles. These factors include: ER at biopsy (OR=2.18; 95% CI 1.43–3.32; *P <*0.001), pathological type at biopsy (OR=0.51; 95% CI 0.40–0.65; *P <*0.001), baseline platelet (OR=1.45; 95% CI 1.02–2.05; *P* =0.037), preoperative PLR (OR=1.63; 95% CI 1.0–2.64; *P* =0.046). Incorporate the above factors as shown in **Figure 3**.

### Univariate analysis of ER status

The proportion of ER status patients with different clinical and hematological parameters is shown in **Table 4**. In univariate logistic regression, among ER-negative patients, 93% had PR < 1% (*P <*0.001),71% had zero HER2 status (*P <*0.001),64.3% had invasive ductal carcinoma (*P <*0.001) and 78% had Ki67 > 14% (*P <*0.001). The proportion of LuminalA 279patients reached 94% (*P <*0.001) and nearly 53% achieved pCR (*P <*0.001). However, there was no statistical difference between ER status in terms of age, baseline and preoperative hematologic parameters (*P* >0.05).

**Table 4:** Univariate analysis for ER status.

Characteristics	Overall	ER-negative	ER-positive	*p*
	998	272	726	
Age				
≦50	529 (53.0)	133 (48.9)	396 (54.5)	0.128
>50	469 (47.0)	139 (51.1)	330 (45.5)	
PR				
<1%	384 (38.5)	253 (93.0)	131 (18.0)	<0.001
≧1%	614 (61.5)	19 (7.0)	595 (82.0)	
HER2				
0	530 (53.1)	193 (71.0)	337 (46.4)	<0.001
1+	413 (41.4)	70 (25.7)	343 (47.2)	
2+/FISH-	55 (5.5)	9 (3.3)	46 (6.3)	
Pathology Type				
Invasive ductal carcinoma	778 (78.0)	175 (64.3)	603 (83.1)	<0.001
Invasive breast cancer	189 (18.9)	83 (30.5)	106 (14.6)	
Specific breast cancer	9 (0.9)	3 (1.1)	6 (0.8)	
unknow	22 (2.2)	11 (4.0)	11 (1.5)	
Histological Grade				
I	41 (4.1)	4 (1.5)	37 (5.1)	<0.001
II	615 (61.6)	93 (34.2)	522 (71.9)	
III	104 (10.4)	71 (26.1)	33 (4.5)	
unknow	238 (23.8)	104 (38.2)	134 (18.5)	
Ki67				
≦14%	276 (27.7)	79 (22.3)	197 (30.6)	0.006
>14%	722 (72.3)	276 (77.7)	446 (69.4)	
Molecular subtype				
Luminal A	259 (26.0)	256 (94.1)	3 (0.4)	<0.001
Luminal B	296 (29.7)	0 (0.0)	296 (40.8)	
TNBC	443 (44.4)	16 (5.9)	427 (58.8)	
Axillary node pathologic state				
pCR	355 (35.6)	143 (52.6)	212 (29.2)	<0.001
Non-pCR	643 (64.4)	129 (47.4)	514 (70.8)	
Baseline lymphocyte				
low	438 (43.9)	114 (41.9)	324 (44.6)	0.485
high	560 (56.1)	158 (58.1)	402 (55.4)	
Baseline neutrophil				
low	194 (19.4)	62 (22.8)	132 (18.2)	0.121
high	804 (80.6)	210 (77.2)	594 (81.8)	
Baseline monocyte				
low	279 (28.0)	64 (23.5)	215 (29.6)	0.068
high	719 (72.0)	208 (76.5)	511 (70.4)	
Baseline platelet				
low	273 (27.4)	72 (26.5)	201 (27.7)	0.761
high	725 (72.6)	200 (73.5)	525 (72.3)	
Baseline NLR				
low	151 (15.1)	48 (17.6)	103 (14.2)	0.208
high	847 (84.9)	224 (82.4)	623 (85.8)	
Baseline PLR				
low	377 (37.8)	105 (38.6)	272 (37.5)	0.797
high	621 (62.2)	167 (61.4)	454 (62.5)	
Baseline LMR				
low	776 (77.8)	211 (77.6)	565 (77.8)	1
high	222 (22.2)	61 (22.4)	161 (22.2)	
Baseline SII				
low	452 (45.3)	130 (47.8)	322 (44.4)	0.368
high	546 (54.7)	142 (52.2)	404 (55.6)	
Preoperative lymphocyte				
low	677 (67.8)	178 (65.4)	499 (68.7)	0.36
high	321 (32.2)	94 (34.6)	227 (31.3)	
Preoperative neutrophil				
low	562 (56.3)	152 (55.9)	410 (56.5)	0.923
high	436 (43.7)	120 (44.1)	316 (43.5)	
Preoperative monocyte				
low	717 (71.8)	192 (70.6)	525 (72.3)	0.645
high	281 (28.2)	80 (29.4)	201 (27.7)	
Preoperative platelet				
low	797 (79.9)	209 (76.8)	588 (81.0)	0.171
high	201 (20.1)	63 (23.2)	138 (19.0)	
Preoperative NLR				
low	784 (78.6)	210 (77.2)	574 (79.1)	0.582
high	214 (21.4)	62 (22.8)	152 (20.9)	
Preoperative PLR				
low	94 (9.4)	30 (11.0)	64 (8.8)	0.345
high	904 (90.6)	242 (89.0)	662 (91.2)	
Preoperative LMR				
low	273 (27.4)	75 (27.6)	198 (27.3)	0.988
high	725 (72.6)	197 (72.4)	528 (72.7)	
Preoperative SII				
low	566 (56.7)	155 (57.0)	411 (56.6)	0.973
high	432 (43.3)	117 (43.0)	315 (43.4)	

## Discussion

To our knowledge, this is the first study demonstrating hematological parameters prognostic significance in HER2-low breast cancer patients treated with NACT. This study first showed that baseline platelet, preoperative PLR was an independent predictor of axillary pCR. whereas the patient’s axillary pCR was not substantially correlated with the NLR, MLR and SII.

Several studies have investigated the pCR-predictive roles of systemic immune-infammatory markers (SIMs), but there was no consensus on the optimal indicator. As BC is a heterogeneous disease, different subtypes are amenable to different therapies (18). This study focused on patients with HER-low BC treated with neoadjuvant chemotherapy to avoid the bias among subtlety and find the optimal predictors in this subgroup. For early-stage breast cancer patients who are eligible for surgery, NAT has become the standard treatment method. However, evaluating treatment efficacy relies on pathological assessment of surgical specimens, and this opportunity for evaluation is only available once. Therefore, it is of great significance to search for indicators that can infer pathological response without the need for surgery (19).

Based on the traditional evaluation of neoadjuvant therapy effect, pathological and imaging methods, which rely on the limitation of the patient’s chemotherapy cycle time, we consider whether the efficacy of the patient’s neoadjuvant chemotherapy cycle can be predicted in advance through the indicators commonly used in clinical cases to supplement the pathological diagnosis.

The clinicopathological characteristics of cancer patients and SIMs derived from the quantification of immune and inflammatory cells in peripheral blood have the potential to serve as predictors for pathological treatment response. In the present study, ER status, clinical Pathology Type. The level of platelets before neoadjuvant chemotherapy and the ratio of platelets to lymphocytes before surgery were independent predictors of pCR in patients with HER2-low BC treated with NAT.

Qijun Zheng MD et al. (8) study included 953 and 267 patients from Peking University Cancer Hospital and Peking University First Hospital, respectively. In the construction cohort, 39.7% (238 of 600) of patients achieved axillary pCR after NAC. The result of multivariate logistic regression analysis showed that tumor grade, NAC regimen, and tumor biologic subtype were significant independent predictors of ypN0 (*P* < 0.05). This study found that the pCR rate reached 35% similar to our results, however in terms of tumor grade, and tumor biologic subtype, we didn’t find any of these factors were significant independent predictors of pCR, we considered this to be related to HER2 status, sample size, and selection bias, and statistical confirmation was needed at a later stage. Many previous studies have investigated systemic immune-inflammatory markers before NAT. Some studies have also suggested that a combination of systemic immune-inflammatory markers may better predict pCR in breast cancer patients after NAT (20, 21). Rulan Ma et al. (22) study found that white blood cell (WBC) platelet (PLT), PLR were independent predictors of pCR after NAC. There is also study found that PLR is an independent predictor of pCR after NAT in HER2-low breast cancer (23), This is consistent with our conclusions.

Some studies showed how lymphopenia can be a predictor of poor outcome in BC patients with increased risk of disease progression and worse long-term survival, this is associated with a weaker anti-tumor response and a lower number of tumor-infiltrating lymphocytes (TILs) (24, 25). The circulating lymphocyte count and lymphocytes characteristics, especially T-cell receptor diversity, have been investigated, either alone or in combination, as prognostic factors at diagnosis in BC patients (26). However, our study found no correlation between baseline peripheral blood lymphocyte count and preoperative peripheral blood lymphocyte count and the incidence of axillary pCR. Considering that this difference may be characteristic of the HER2-low BC subtype, the molecular mechanism of HER2-low BC and lymphocyte infiltration can be further investigated.

At the same time, we evaluated the influencing factors of axillary pCR in ER-positive and ER-negative patients, and found that pCR was more easily achieved in ER-negative patients, which was similar to the result of He et al. (27). However such patients tended to have poor prognosis. In addition, we found no correlation between ER status and hematological parameters.

However, the predictive roles of these markers in patients with HER2-low BC receiving NAT remain unclear. This study retrospectively analyzed the registered data of patients with HER2-low BC who were treated with NAC This is the first study to comprehensively assess the roles of clinicopathological characteristics, hematologic markers, NLR, PLR, LMR and SII in predicting axillary pCR in patients with HER2-low BC treated with NAC. Other data that may affect the treatment efficacy were also analyzed. Our results showed higher baseline PLT, and preoperative PLR was associated with a higher incidence of pCR. Some studies have suggested that the number of circulating platelets is associated with the level of serum VEGF-A and that platelet release promotes tumor growth and angiogenesis via VEGF integrins cooperative signaling in animal models of breast cancer (28, 29). In addition, it has been shown that platelets promote tumor proliferation via adenosine diphosphate receptors or metalloproteinase-9 and that their inhibitors reduce platelet-enhanced cancer cell proliferation (30, 31). Based on these results, it can be hypothesized that as the baseline PLT and preoperative PLR increases, the oncological outcomes of patients with cancer better.

This study has several limitations. First, this was a single-institute retrospective study, thus, selection bias may have occurred. Second, the patients included in this study was relatively Prognostic information is missing. Third, Pathology Type Histological Grade Partial deletion is present, and Invasive ductal carcinoma (IDC) accounts for almost 80% of patients. Future prospective large-scale studies, including a more varied population with a longer follow-up period are warranted to verify the outcomes of this study.

## Conclusion

In conclusion, our study found that ER, pathology type, baseline PLT and preoperative PLR have independent predictive value for pCR after NAT in HER2-low breast cancer. However, future prospective studies are warranted to verify the predictive value of Those factors.

## Data Availability

The original contributions presented in the study are included in the article/**Supplementary Material**. Further inquiries can be directed to the corresponding authors.
